# Retraction: PER-TIM Interactions with the Photoreceptor Cryptochrome Mediate Circadian Temperature Responses in *Drosophila*

**DOI:** 10.1371/journal.pbio.1002403

**Published:** 2016-02-25

**Authors:** Rachna Kaushik, Pipat Nawathean, Ania Busza, Alejandro Murad, Patrick Emery, Michael Rosbash

Our original paper strongly implicated the photoreceptor cryptochrome (CRY) in the response of the *Drosophila* circadian system to temperature. The claim was based on three findings:

1. CRY formed a complex with the two key clock proteins Period (PER) and Timeless (TIM) not only in response to a light pulse but also in response to a 37°C heat pulse.
2. A mutant strain missing wild-type CRY did not phase shift in response to a heat pulse, implicating CRY in heat-mediated phase shifts in addition to its well-known importance for light-mediated phase shifts. This mutant phenotype was rescued by expressing wild-type CRY within clock neurons.
3. The absence of CRY inhibited the *per* ^*L*^ effect on another temperature-centric feature of rhythms, temperature compensation of circadian period. The period of *per* ^*L*^ becomes longer at elevated temperatures, whereas it is essentially unaffected in wild-type flies. We reported that a double mutant strain, expressing PERL and also missing wild-type CRY, recovered normal temperature compensation. In other words, CRY was necessary for PERL to affect temperature compensation, consistent with a PER-CRY interaction.

We were contacted some months ago by Mike Young and colleagues at The Rockefeller University, who could not repeat the second finding. We subsequently repeated this experiment ourselves and also observed what Young and colleagues told us, i.e., we could not replicate the original observation that implicated CRY in heat-mediated phase shifts. As we no longer had the double mutant strain necessary to repeat the third finding (restoration of proper temperature compensation in a double mutant strain containing the mutant perL chromosome and missing wild-type CRY), we constructed a very similar strain and assayed these *per* ^*L*^; *cry* ^*0*^ flies for temperature compensation. It was aberrant, essentially indistinguishable from single mutant *per* ^*L*^ flies. We have therefore failed to reproduce two of the three findings in the original paper.

Although we have absolutely no explanation for the discrepancies with the original results and despite the lack of an attempt to reproduce the first finding (the heat-mediated formation of a CRY-PER-TIM interaction), the failure to reproduce two of the three findings in the original paper compels us to retract the paper. We deeply regret any inconvenience that this has caused the scientific community.
